# Corneal confocal microscopy detects small nerve fibre damage in patients with painful diabetic neuropathy

**DOI:** 10.1038/s41598-020-60422-7

**Published:** 2020-02-25

**Authors:** Alise Kalteniece, Maryam Ferdousi, Shazli Azmi, Womba M. Mubita, Andrew Marshall, Giuseppe Lauria, Catharina G. Faber, Handrean Soran, Rayaz A. Malik

**Affiliations:** 1 Division of Cardiovascular Sciences, Cardiac Centre, Faculty of Medical and Human Sciences, University of Manchester and NIHR/Wellcome Trust Clinical Research Facility, Manchester, UK; 2 Weill Cornell Medicine-Qatar, Qatar Foundation, Education City, Doha, Qatar; 3 Cardiovascular Trials Unit, Central Manchester University Hospital NHS Foundation Trust, Manchester, UK; 4 Neuroalgology Unit and Skin Biopsy, Peripheral Neuropathy and Neuropathic Pain Center, IRCCS Foundation “Carlo Besta” Neurological Institute, Milan, Italy; 5 Department of Neurology, School of Mental Health and Neuroscience, Maastricht University Medical Center, Maastricht, The Netherlands

**Keywords:** Diabetes, Neuropathic pain

## Abstract

Neuropathic pain is believed to arise from damage to nociceptive C fibres in diabetic neuropathy (DN). We have utilised corneal confocal microscopy (CCM) to quantify the severity of small nerve fibre damage in relation to the severity of neuropathic pain and quality of life (QoL) in patients with and without painful DN. 30 controls and patients with painful (n = 78) and painless (n = 62) DN underwent assessment of large and small nerve fibre function, CCM, neuropathic symptoms (small fibre neuropathy symptom inventory questionnaire, neuropathic pain scale) and QoL (SF-36, pre-R-ODS and hospital anxiety and depression scale). Patients with painful compared to painless DN, had comparable neurophysiology and vibration perception, but lower corneal nerve fibre density (20.1 ± 0.87 vs. 24.13 ± 0.91, P = 0.005), branch density (44.4 ± 3.31 vs. 57.74 ± 3.98, P = 0.03), length (19.61 ± 0.81 vs. 22.77 ± 0.83, P = 0.01), inferior whorl length (18.03 ± 1.46 vs. 25.1 ± 1.95, P = 0.005) and cold sensation threshold (21.35 ± 0.99 vs. 26.08 ± 0.5, P < 0.0001) and higher warm sensation threshold (43.7 ± 0.49 vs. 41.37 ± 0.51, P = 0.004) indicative of small fibre damage. There was a significant association between all CCM parameters and the severity of painful neuropathic symptoms, depression score and QoL. CCM identifies small nerve fibre loss, which correlates with the severity of neuropathic symptoms and reduced QoL in patients with painful diabetic neuropathy.

## Introduction

Pain is one of the most disturbing symptoms that accompany diabetic peripheral neuropathy and can critically affect the patient’s quality of life in about one-third of patients with diabetic neuropathy^1,2^. The true prevalence of painful diabetic neuropathy (DN) may be even higher, as most patients either do not report their symptoms or attribute them to diabetes^3^ and there is disagreement between the patient’s and physician’s perception of pain^4^. Neuropathic pain usually manifests as “tingling”, “pins and needles”, deep and dull aching, burning or electrical sensation in the feet and lower limbs, with a reduced ability to perform daily activities, nocturnal exacerbation with disturbed sleep resulting in lower mood, anxiety, depression and reduced quality of life^1^.

The underlying mechanisms of painful DN are complex and include distal small fibre degeneration with regeneration^5^, autonomic dysfunction^6^, impaired skin microcirculation^7^ and epineurial blood flow^8^, altered sodium^9^ and calcium channel expression^10^, central sensitization^11^ and alterations in the spinal cord and brain^12,13^.

The diagnosis of painful DN is challenging and subjective, based on establishing neurologic deficits and identifying the type and severity of pain through validated questionnaires^14^ including neuropathic pain scale (NPS)^15^, small fibre neuropathy symptom inventory questionnaire (SFN-SIQ)^16^ and quality of life (QoL) through the hospital anxiety and depression scale (HADS)^17^ and the 36-item short form health survey (SF-36)^18^. Although, pain questionnaires are easy to administer, they have limited sensitivity and low reliability^19^. Objective tests such as nerve conduction studies, quantitative sensory testing (QST), skin punch biopsy and CCM can help to define underlying nerve damage. Two large neuropathy phenotyping studies have shown that nerve conduction studies do not differentiate painful from painless neuropathy^20^, although thermal thresholds were associated with the severity of painful diabetic neuropathy^21^. With regard to structural damage to small fibres, reduced intraepidermal nerve fibre length^22^ and increased regeneration and axonal swellings have been found in patients with painful DN^23^. Corneal confocal microscopy (CCM) is a rapid non-invasive surrogate marker for small nerve fibre damage in diabetic peripheral neuropathy^24^. In two small studies we and others showed greater corneal nerve fibre loss in the central cornea^22,25^ and inferior whorl^26^ in patients with painful compared to painless diabetic neuropathy.

This study aimed to establish if CCM can differentiate patients with painful compared to painless DN and whether the severity of corneal nerve damage was associated with the severity of neuropathic pain and quality of life.

## Results

### Demographic and clinical findings

Controls were age-matched with the patients with diabetes mellitus. Blood pressure (BP) was comparable between groups. The duration of diabetes was comparable between patients with painless and painful DN. 58% of patients with PDN were on pain medications. BMI (P < 0.005) and HbA1c (P < 0.0001) were significantly higher and total cholesterol (P < 0.0001), LDL (P < 0.05) and HDL (P < 0.04) were significantly lower in patients with painful DN compared to healthy controls. Patients with painless DN had a significantly higher HbA1c (P < 0.0001) and lower total cholesterol (P < 0.0001) compared to healthy controls. Total cholesterol (P = 0.03) and triglycerides (P = 0.01) were significantly higher in painful compared to painless DN (Table 1).

**Table 1:** Clinical demographics, peripheral neuropathy assessment and CCM in patients with painless and painful diabetic neuropathy.

	Controls (n = 30)	Painless DN (n = 62)	Painful DN (n = 78)
Age	61.2 ± 1.33	66.09 ± 1.1	64.09 ± 1.16
Gender (female/male)	16/14	18/44	30/48
Duration of diabetes (years)	N/A	23.67 ± 2.5	19.86 ± 1.6
T1DM/T2DM	N/A	24/38	17/61
BP Systolic/ Diastolic (mmHg)	133.7 ± 2.7/73.4 ± 1.8	130.4 ± 3.2/66.8 ± 1.5	132.72 ± 2.44/71.8 ± 2.3
BMI (kg/m^2^)	27.6 ± 1.02	30.2 ± 1.12	33.1 ± 1.07^
Total cholesterol (mmol/L)	5.1 ± 0.19	3.77 ± 0.1^	4.18 ± 0.1^*
HDL (mmol/L)	1.61 ± 0.08	1.42 ± 0.07	1.34 ± 0.05^
LDL (mmol/L)	2.77 ± 0.13	2.04 ± 0.3	1.98 ± 0.09^
Triglycerides (mmol/L)	1.63 ± 0.14	1.36 ± 0.12	1.92 ± 0.14*
HbA1c (mmol/mol)	38.04 ± 0.69	56.86 ± 2.17^	59.8 ± 1.73^
HbA1c (%)	5.63 ± 0.06	7.35 ± 0.19^	7.62 ± 0.15^
Sural velocity (m/s)	49.16 ± 0.9	39.97 ± 0.81^	40.57 ± 0.77^
Sural amplitude (μV)	16.08 ± 1.56	5.77 ± 0.68^	5.95 ± 0.67^
Cold threshold (°C)	27.67 ± 0.36	26.08 ± 0.5	21.35 ± 0.99^*
Warm threshold (°C)	38.24 ± 0.73	41.37 ± 0.51^	43.7 ± 0.49^*
VPT (V)	8.17 ± 1.06	21.93 ± 1.47^	24.08 ± 1.35^
NDS (0-10)	1.0 ± 0.3	5.89 ± 0.29^	6.79 ± 0.26^*
CNFD (no./mm^2^)	32.58 ± 1.26	24.13 ± 0.91^	20.1 ± 0.87^*
CNBD (no./mm^2^)	90.98 ± 5.4	57.74 ± 3.98^	44.4 ± 3.31^*
CNFL (mm/mm^2^)	26.63 ± 0.98	22.77 ± 0.83^	19.61 ± 0.81^*
IWL (mm/mm^2^)	35.19 ± 2.29	25.14 ± 1.95^	18.03 ± 1.46^*

All data are presented as mean ± SE. HDL – high-density lipoprotein cholesterol, LDL – low-density lipoprotein cholesterol, VPT – vibration perception threshold, NDS – neuropathy disability score, CNFD – corneal nerve fibre density, CNBD – corneal nerve fibre branch density, CNFL – corneal nerve fibre length, IWL – inferior whorl length. ^P < 0.05 compared to controls, *P < 0.05 compared to painless.

### Neuropathy severity, neurophysiology and QST

NDS (6.79 ± 0.26 vs 5.89 ± 0.29 vs 1.0 ± 0.3, P < 0.0001) was significantly higher in painful and painless DN compared to healthy controls and was also significantly higher in painful compared to painless DN (P = 0.05). Sural nerve conduction velocity (40.57 ± 0.77 vs 39.97 ± 0.81 vs 49.16 ± 0.9, P < 0.0001) and amplitude (5.95 ± 0.67 vs 5.77 ± 0.68 vs 16.08 ± 1.56, P < 0.0001) were significantly lower and VPT (24.08 ± 1.35 vs 21.93 ± 1.47 vs 8.17 ± 1.06, P < 0.0001) was significantly higher in patients with painful and painless DN compared to healthy controls, but did not differ between patients with painful and painless DN. WT (43.7 ± 0.49 vs 41.37 ± 0.51 vs 38.24 ± 0.73, P < 0.0001) was significantly higher in patients with painful and painless DN compared to healthy controls and differed between painful and painless DN (P = 0.004). CT (21.35 ± 0.99 vs 26.08 ± 0.5 vs 27.67 ± 0.36) was lower in painful DN compared to painless DN (P < 0.00001) and controls (P < 0.0001) (Table 1).

### Corneal confocal microscopy

**Figure 1:**
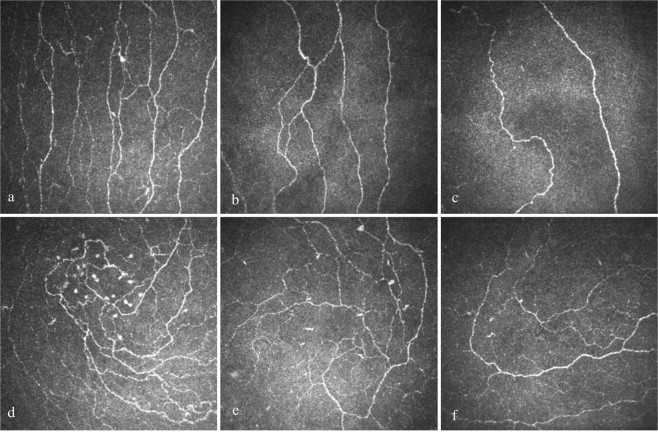
CCM images of the central cornea and inferior whorl in a healthy control (**a**,**d**), patient with painless DN (**b**,**e**) and patient with painful DN (**c**,**f**).

**Figure 2:**
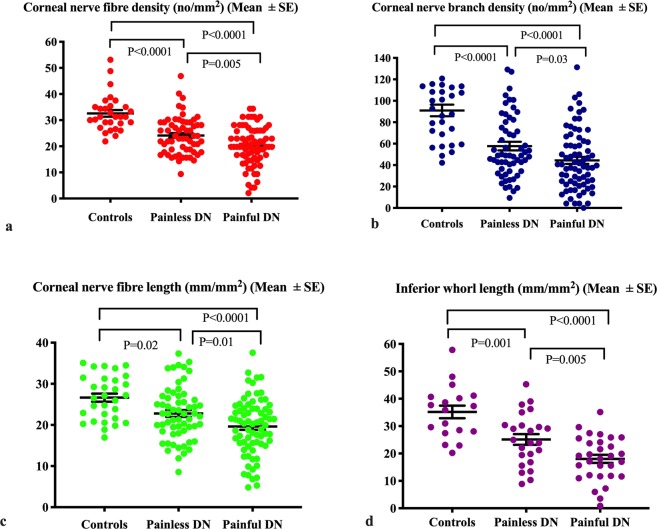
Dot plots of corneal nerve fiber parameters (mean ± SE): (**a**) corneal nerve fiber density (CNFD), (**b**) corneal nerve branch density (CNBD), (**c**) corneal nerve fiber length (CNFL), (**d**) inferior whorl length (IWL) in healthy controls and patients with painless and painful DN.

All CCM parameters were significantly lower in painless and painful DN compared to healthy controls: CNFD (24.13 ± 0.91, P < 0.0001 and 20.1 ± 0.87, P < 0.0001), CNBD (57.74 ± 3.98, P < 0.0001 and 44.4 ± 3.31, P < 0.0001), CNFL (22.77 ± 0.83, P = 0.02 and 19.61 ± 0.81, P < 0.0001) and IWL (25.14 ± 1.95, P = 0.001 and 18.03 ± 1.46, P < 0.0001) (Table 1, Figs. 1 and 2). In addition, CNFD (P = 0.005), CNBD (P = 0.03), CNFL (P = 0.01) and IWL (P = 0.005) were lower in patients with painful compared to painless DN (Table 1).

### Association between CCM and quality of life

There were weak but significant correlations between the VAS score and CNFD (r = −0.2, P = 0.02), CNBD (r = −0.2, P = 0.02) and IWL (r = −0.4, P = 0.02). Also, there were significant positive correlations between the average SF-36 score with CNFD (r = 0.2, P = 0.01), CNBD (r = 0.3, P = 0.002), CNFL (r = 0.3, P = 0.01) and IWL (r = 0.5, P = 0.002) and significant negative correlations between the severity of depression measured using the HADS questionnaire with CNFD (r = −0.2, P = 0.01), CNBD (r = −0.3, P = 0.003), CNFL (r = −0.3, P = 0.008) and IWL (r = −0.5, P = 0.003). Significant inverse correlations were found between the severity of neuropathic symptoms assessed using the SFN-SIQ questionnaire with CNFD (r = −0.2, P = 0.01), CNBD (r = −0.3, P = 0.007), CNFL (r = −0.2, P = 0.01) and IWL (r = −0.5, P = 0.002). With the pre-R-ODS questionnaire, there were significant positive correlations between the dimensions of carry, move, handle the object with CNFD (r = 0.2, P = 0.04) and IWL (r = 0.3, P = 0.03). Walking and movement correlated significantly with CNFD (r = 0.2, P = 0.01), CNBD (r = 0.2, P = 0.01), CNFL (r = 0.2, P = 0.03) and IWL (r = 0.4, P = 0.01) and between self-care with CNFD (r = 0.2, P = 0.04) and between eating and CNFD (r = 0.3, P = 0.005), CNBD (r = 0.2, P = 0.01) and CNFL (r = 0.3, P = 0.004). There was a correlation between household activities with CNBD (r = 0.2, P = 0.02) and CNFL (r = 0.2, P = 0.04) and between IWL and daily tasks (r = 0.3, P = 0.03) and the ability to change and hold body position (r = 0.3, P = 0.02).

### Neuropathic pain and QOL

**Figure 3:**
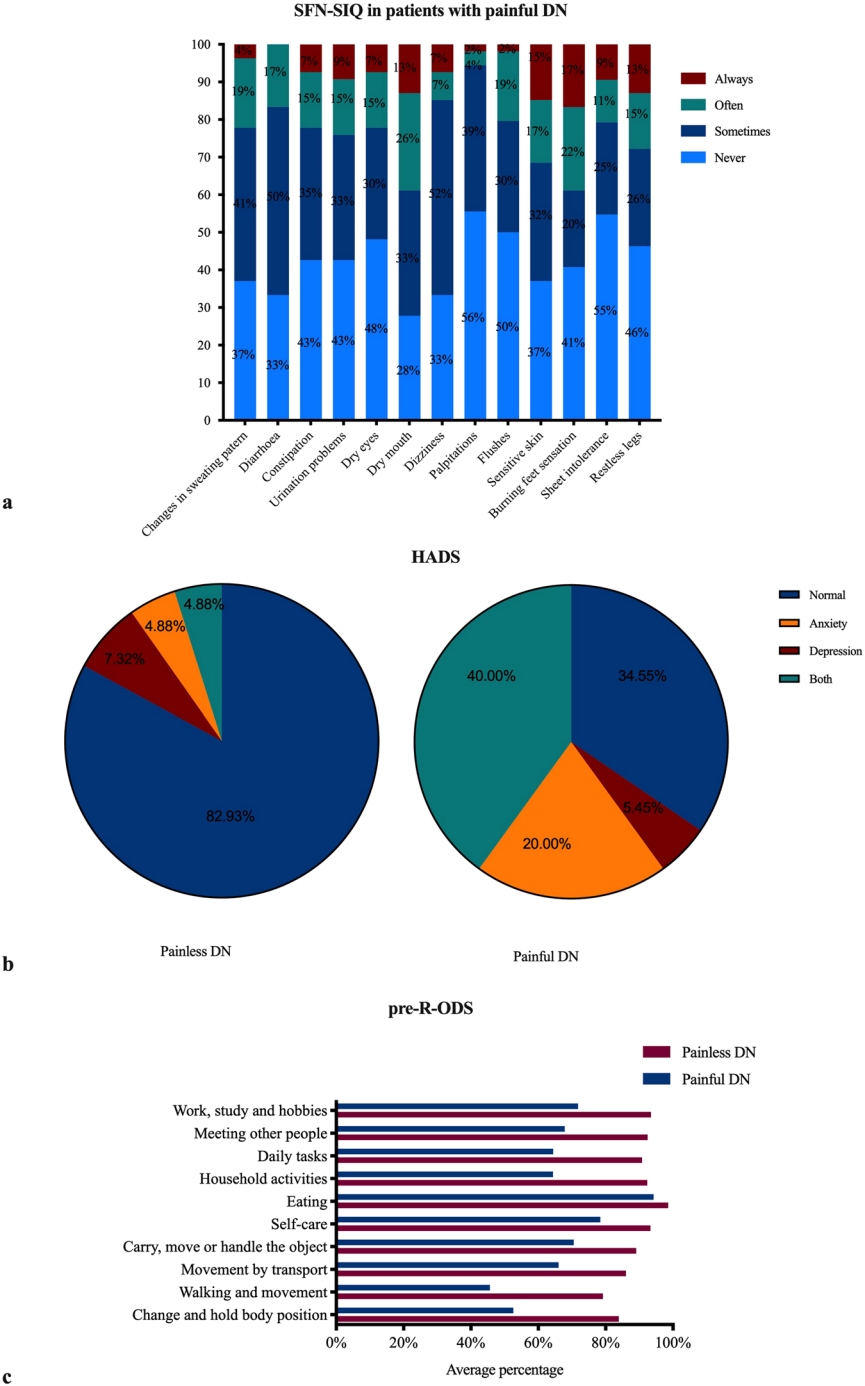
Measures of QoL and pain. (**a**) Small fibre neuropathy symptom inventory questionnaire (SFN-SIQ) in patients with painful DN; (**b**) Hospital anxiety depression scale (HADS); (**c**) preliminary Rasch-built Overall Disability Scale (pre-R-ODS) in patients with painless and painful DN.

The most common descriptors of neuropathic pain among patients with painful DN assessed with the NPS questionnaire were: ~95% suffered from unpleasantness and intensity, ~89% reported deep pain, ~70% reported itchiness and ~65% cold pain. Almost every individual experienced more than one type of pain. Based on the SFN-SIQ questionnaire, the most common symptoms among patients with painful DN were dry mouth (~72%), dizziness (~67%), diaorrhea (~67%) and sensitive skin (~63%) (Fig. 3a). 83% of patients with dry mouth symptoms and 80% of patients with dizziness were on pain medications.

Patients with painful DN had a significantly reduced QoL compared to painless DN based on the SF-36 score (47.19 ± 2.92 vs. 77.04 ± 2.66, P < 0.0001). The HADS depression (7.23 ± 0.53 vs. 3.78 ± 0.51, P < 0.0001) and anxiety score (7.89 ± 0.56 vs. 4.09 ± 0.45, P < 0.0001) was higher in patients with painful compared to painless DN (Table 2). Based on HADS, 20% (11/55) of patients with painful DN and approximately 5% (2/41) of patients with painless DN suffered from anxiety, while 5.5% (3/55) of patients with painful DN and 7% (3/41) of patients with painless DN suffered from depression, however, 40% (22/55) of patients with painful DN and 5% (2/41) of patients with painless DN suffered from both – depression and anxiety, respectively (Fig. 3b). Patients with painful DN on treatment for neuropathic pain had significantly worse depression (P = 0.002) and anxiety scores (P = 0.05), SFN-SIQ (P < 0.0001) and quality of life (SF-36) (P = 0.003) (Supplementary Table S1).

**Table 2:** Pain and Quality of Life in patients with painless and painful diabetic neuropathy.

	Painless DN (n = 41)	Painful DN (n = 55)
HADS depression (0-21)	3.78 ± 0.51	7.23 ± 0.53*
HADS anxiety (0-21)	4.09 ± 0.45	7.89 ± 0.56*
SFN-SIQ (0-39)	5.13 ± 0.65	11.61 ± 0.91*
SF-36 (0-100)	77.04 ± 2.66	47.19 ± 2.92*
Pre-R-ODS (%)		
• Change and hold body position (%)	83.93 ± 3.33	52.56 ± 3.38*
• Walking and movement (%)	79.19 ± 4.21	45.65 ± 3.52*
• Movement by transport (%)	86.01 ± 3.27	66.02 ± 3.26*
• Carry, move, handle the objects (%)	89.07 ± 2.99	70.53 ± 3.08*
• Self-care (%)	93.29 ± 2.14	78.39 ± 2.92*
• Eating (%)	98.57 ± 1.42	94.24 ± 1.93
• Household (%)	92.36 ± 2.42	64.38 ± 4.21*
• Daily tasks (%)	90.79 ± 2.81	64.4 ± 3.69*
• Meeting other people (%)	92.47 ± 3.22	67.87 ± 4.09*
• Work, study, hobbies (%)	93.49 ± 2.04	71.81 ± 3.43*

All the data are presented as mean ± SE. HADS – hospital anxiety and depression scale; SFN-SIQ – small fibre neuropathy symptom inventory questionnaire; SF-36 – 36-item short form health survey; Pre-R-ODS – preliminary Rasch-built Overall Disability Scale. * P < 0.05 compared to painless.

QoL was assessed using the pre-R-ODS questionnaire (Table 2, Fig. 3c). Almost all aspects of QoL were significantly worse in patients with painful compared to painless DN: change and hold body position (52.56 ± 3.38 vs. 83.93 ± 3.33, P < 0.0001), walking and movement (45.65 ± 3.52 vs. 79.19 ± 4.21, P < 0.0001), movement by transport (66.02 ± 3.26 vs. 86.01 ± 3.27, P < 0.0001), carry, move, handle object (70.53 ± 3.08 vs. 89.07 ± 2.99, P < 0.0001), self-care (78.39 ± 2.92 vs. 93.29 ± 2.14, P < 0.0001), household (64.38 ± 4.21 vs. 92.36 ± 2.42, P < 0.0001), daily tasks (64.4 ± 3.69 vs. 90.79 ± 2.81, P < 0.0001), meeting people (67.87 ± 4.09 vs. 92.47 ± 3.22, P < 0.0001) work, study, hobbies (71.81 ± 3.43 vs. 93.49 ± 2.04, P < 0.0001).

## Discussion

Painful diabetic neuropathy has a major impact on the patient’s quality of life as a result of associated anxiety and depression^27^. The exact cause of neuropathic pain remains elusive^28^, especially as pain can be experienced when there is little or no existing damage to nerves and a patient with severe neuropathy may not have any complaints^29^. Previous studies have reported that damage to Aδ and C fibres is associated with neuropathic pain^30,31^. CCM can quantify small fibre pathology and stratify the severity of DN^32^. In the present study, we demonstrate greater small fibre dysfunction (warm and cold thresholds) and damage to corneal nerve fibres but no difference in large fibre measures such as VPT and nerve conduction studies in patients with painful compared to painless DN. This agrees with our recent study showing reduced corneal nerve parameters in patients with painful compared to painless DN and an association with the severity of pain^26^.

Quattrini *et al*.^7^ previously reported a greater reduction in corneal nerve fibre length and intra-epidermal nerve fibre length in patients with painful compared to painless DN^22^. Wang *et al*.^25^ reported significant alterations in corneal nerve morphology and a direct correlation with the severity of pain^25^, whereas Cheng *et al*.^23^ reported axonal swelling in patients with painful compared to painless DN^23^.

This study also demonstrates a significant reduction in cold sensation threshold and an increase in warm sensation threshold in patients with painful compared to painless DN, consistent with Raputova *et al*.^21^ who reported significantly worse cold and warm detection thresholds in patients with mild and moderate to severe neuropathic pain compared to patients without neuropathic pain^21^. Themistocleous *et al*.^20^ reported a significant abnormality in QST parameters in patients with neuropathic pain but found no difference in nerve conduction studies, intraepidermal nerve fibre density and cold pain threshold^20^. The current study strengthens the evidence of greater small fibre loss and dysfunction in patients with painful compared to painless neuropathy.

Validated questionnaires are an important tool for the assessment of neuropathic pain and its effect on the patients quality of life^33^, allowing us to assess the effect on patients’ lives including their mood, daily activities and social life. We show a significant correlation between measures of corneal nerve damage, especially at the inferior whorl with the presence and severity of neuropathic pain, quality of life and severity of depression. However, this weak association should be interpreted cautiously and suggests that factors other than the extent of small fibre damage contribute to a worse QoL and severity of pain. Patients with painful DN also suffered from greater anxiety and depression compared to those with painless DN. Previously, Gore *et al*.^34^ also demonstrated higher scores of anxiety and depression in patients with more severe neuropathic pain^34^. Dizziness and dry mouth were the most common symptoms among patients with painful DN and could be attributed to concomitant use of medications to relieve neuropathic pain. The most common type of pain reported was deep pain, whilst the least common was itchy pain. Others using NPS have also reported sharp and deep pain to be common whilst itching and cold pain were infrequent^1,35^. Using the pre-R-ODS questionnaire we show a significant reduction in all dimensions of QoL except eating. The SF-36 average score indicated a significant reduction in QoL (e.g. physical functioning and health, wellbeing, emotional problems and general health) in patients with painful compared to painless DN.

To our knowledge this is the first study to evaluate the association between the severity of small fibre neuropathy using CCM and its relationship to the severity of painful symptoms, patient mood, daily activities, social life and overall QoL. Although the VAS is a validated scale for the assessment of the severity of pain, we acknowledge it is very subjective. Another limitation of the current study is the lack of validated neuropathy specific QoL tools such as NeuroQoL and Norfolk QoL-DN that more accurately quantify QoL in diabetic neuropathy.

In conclusion, we show that corneal confocal microscopy detects greater corneal nerve fibre loss in patients with painful diabetic neuropathy and this correlates with the severity of neuropathic pain, the patient’s mood and QoL. These data encourage further studies to assess the potential utility of CCM in relation to a change in the severity of neuropathic pain and quality of life in clinical intervention trials.

## Methods

### Study subjects

140 patients with type 1 (n = 41) and type 2 diabetes (n = 99) and 30 age-matched healthy controls were studied. All participants underwent demographic and clinical examination, detailed assessment of peripheral neuropathy and ophthalmic examination. Participants with a history of malignancy, vitamin B12 or folate deficiency, chronic renal and/or liver impairment, connective tissue or infectious disease, neuropathy of other cause than diabetes, current diabetic foot ulcer, corneal trauma, or systemic disease of the cornea and contact lens wear were excluded from the study. A signed consent form was obtained from each participant prior to taking part in the study. This study adhered to the tenets of the Declaration of Helsinki and was approved by Greater Manchester East Research Ethics Committee.

### Demographic and clinical neuropathy assessment

BMI, blood pressure, lipid profile and glycated haemoglobin (HbA1c) were measured in each participant. Neurological deficits were measured using the simplified Neuropathy disability score (NDS) (0-10) and patients were confirmed to have DN based on an NDS > 2. Further, based on the visual analogue scale (VAS), patients were divided into two groups of painful (VAS > 4) (n = 78) and painless DN (VAS ≤ 4) (n = 62)^36^.

Vibration perception threshold (VPT) was measured with a Neurothesiometer (Scientific Laboratory Supplies, Wilford, Nottingham, UK), and cold and warm perception thresholds were assessed with the neurosensory analyzer (TSA-II NeuroSensory Analyzer, Medoc Ltd., Ramat-Yishai, Israel) on the dorsum of the left foot. In addition, sural sensory nerve amplitude and nerve conduction velocity were assessed by a consultant neurophysiologist using nerve conduction testing machine (Dantec “Keypoint” system, Dantec Dynamics Ltd., Bristol, North Somerset, UK).

### Corneal confocal microscopy

All participants were examined using slit-lamp biomicroscopy to eliminate the presence of ocular surface infection, corneal ulceration and narrow angle glaucoma prior to CCM examination. Both eyes were examined with a laser-scanning CCM (Heidelberg Retinal Tomograph-III Rostock Cornea Module, Heidelberg Engineering GmbH, Heidelberg, Germany) using our previously established protocol^37^. Central corneal nerves and the inferior whorl were imaged at the level of sub-basal nerve plexus. CCM provides two-dimensional images with a resolution of 10μm and a size of 384 × 384 pixels.

Six images from the central cornea and four images from the inferior whorl were selected following our established protocols^38,39^. All corneal parameters were quantified using manual purpose-designed software (CCMetrics, M.A. Dabbah, Imaging Science, The University of Manchester, Manchester, UK). Corneal nerve fibre density (CNFD) (no/mm^2^), branch density (CNBD) (no/mm^2^), fibre length (CNFL) (mm/mm^2^) and inferior whorl length (IWL) (mm/mm^2^) were quantified.

### Pain and quality of life questionnaires

QoL was assessed using the validated Short Form 36 Health Survey (SF-36) score, preliminary Rasch-built Overall Disability Scale (pre-R-ODS), Hospital Anxiety Depression Scale (HADS) and small fibre neuropathy and symptom inventory questionnaire (SFN-SIQ). 96 patients completed all questionnaires.

SF-36 consists of 36 questions, divided into 8 dimensions and scored in a range 0-100^18^. Our analysis included a total score, which was an average value of all 8 dimensions.

A 146-item preliminary Rasch-built Overall Disability Scale (pre-R-ODS) was used to assess the different aspects of a patient’s QoL^40^. Each question was scored as 0 – not possible, 1 – possible with effort and 2 – easy to perform. However, in this study, we could not use Rasch model to interpret the pre-R-ODS questionnaire, as the number of participants who completed the questionnaire was less than the number of questions, hence not fulfilling Rasch model requirements. Therefore, the pre-R-ODS questionnaire was divided into ten different dimensions: change and hold body position, walking and movement by transport, carrying, moving, handling objects, self-care, eating, household, daily tasks, meeting other people and work, studies and hobbies. For each patient, the average score for each dimension was established and expressed as a percentage.

HADS was used to score depression and anxiety levels^17^. The questionnaire consisted of 14 items and was divided into 2 subscales: 7 questions for the assessment of depression and 7 questions for anxiety. Each question was scored in a range of 0-3; the higher number represented the more severe symptoms. The subscale score was summed up from each question (range 0-21) and an average depression and anxiety score was compiled.

Other neuropathy symptoms including dry mouth, dizziness, dry eyes, changes in sweating, were assessed using the validated small fibre neuropathy and symptom inventory questionnaire (SFN-SIQ). SFN-SIQ consisted of 13 items. For each item, the scale range was 0-3 where 0=never, 1=sometimes, 2=often and 3=always^16^. For each participant, the overall score for SFN-SIQ was obtained from the sum of all items.

Neuropathic pain scale (NPS), a scale that consists of 10 items, each on a scale ranging from 0-10 was used to assess the most common types of pain among patients with painful DN^15^.

### Statistical analysis

IBM SPSS statistic software (Version 22.0 for Macintosh, IBM Corporation, New York, NY, USA) was used for the analysis. All values were calculated as mean ± standard error (SE). The significance was considered as P < 0.05. To compare values between more than two groups, One-Way Anova *(post-hoc Bonferroni for parametric parameters and LSD for non-parametric parameters) w*as used. Independent-samples T-test was used to compare mean values between two groups (*Mann-Whitney* U test for non-parametric). *Pearson correlation coefficient (Spearman’s rank correlation coefficient for non-parametric)* was measured to evaluate the association between variables. Graphs were made using GraphPad Prism software (Version 7.0c for Macintosh, GraphPad Software, La Jolla California, USA).

## Supplementary information

Supplementary Information.

## Data Availability

The datasets generated and analysed during the study are available from the corresponding author on a reasonable request
