# Supplementary material for: Corneal confocal microscopy detects small nerve fibre damage in patients with painful diabetic neuropathy
Source: Sci Rep. 2020 Feb 25;10:3371. doi: 10.1038/s41598-020-60422-7 (PMC7042367; doi:10.1038/s41598-020-60422-7)
Supplement: Supplementary file 1 — Supplementary Information. [file 41598_2020_60422_MOESM1_ESM.docx]

**Supplementary table S1**

|  | No treatment (n=32) | On treatment (n=46) |
| --- | --- | --- |
| Age | 66.01 ± 1.97 | 62.76 ± 1.41 |
| Duration of diabetes (years) | 20.00 ± 2.62 | 19.77 ± 2.24 |
| CNFD (no./mm^2^) | 21.32 ± 1.25 | 19.16 ± 1.19 |
| CNBD (no./mm^2^) | 43.92 ± 4.49 | 44.78 ± 4.79 |
| CNFL (mm/mm^2^) | 20.57 ± 1.12 | 18.87 ± 1.14 |
| IWL (mm/mm^2^) | 20.34 ± 2.18 | 16.02 ± 1.91 |
| HADS depression (0-21) | 5.46 ± 0.71 | 8.61 ± 0.68* |
| HADS anxiety (0-21) | 6.67 ± 0.80 | 8.84 ± 0.75* |
| SFN-SIQ (0-39) | 7.71 ± 0.96 | 14.73 ± 1.18* |
| SF-36 (0-100) | 56.91 ± 4.47 | 39.68 ± 3.33* |

CCM, small fibre neuropathy symptoms and Quality of Life measures in patients with and without treatment for painful diabetic neuropathy. All the data are presented as Mean ± SE. CNFD – corneal nerve fibre density, CNBD – corneal nerve fibre branch density, CNFL – corneal nerve fibre length, IWL – inferior whorl length. HADS – hospital anxiety and depression scale; SFN-SIQ – small fibre neuropathy symptom inventory questionnaire; SF-36 – 36-item short form health survey; * P<0.05 for patients with and without treatment for painful DPN.
